# The Effects of C-peptide on Type 1 Diabetic Polyneuropathies and Encephalopathy in the BB/Wor-rat

**DOI:** 10.1155/2008/230458

**Published:** 2008-04-16

**Authors:** Anders A. F. Sima, Weixian Zhang, Zhen-guo Li, Hideki Kamiya

**Affiliations:** ^1^Department of Pathology, Wayne State University, Detroit, MI 48201, USA; ^2^Department of Neurology, Wayne State University, Detroit, MI 48201, USA; ^3^Department of Medicine, Nagoya University School of Medicine, Showa-Ku, Nagoya 466-8550, Japan

## Abstract

Diabetic polyneuropathy (DPN) occurs more frequently in type 1 diabetes resulting in a more severe DPN. The differences in DPN between the two types of diabetes are due to differences in the availability of insulin and C-peptide. Insulin and C-peptide provide gene regulatory effects on neurotrophic factors with effects on axonal cytoskeletal proteins and nerve fiber integrity. A significant abnormality in type 1 DPN is nodal degeneration. In the type 1 BB/Wor-rat, C-peptide replacement corrects metabolic abnormalities ameliorating the acute nerve conduction defect. It corrects abnormalities of neurotrophic factors and the expression of neuroskeletal proteins with improvements of axonal size and function. C-peptide corrects the expression of nodal adhesive molecules with prevention and repair of the functionally significant nodal degeneration. Cognitive dysfunction is a recognized complication of type 1 diabetes, and is associated with impaired neurotrophic support and apoptotic neuronal loss. C-peptide prevents hippocampal apoptosis and cognitive deficits. It is therefore clear that substitution of C-peptide in type 1 diabetes has a multitude of effects on DPN and cognitive dysfunction. Here the effects of C-peptide replenishment will be extensively described as they pertain to DPN and diabetic encephalopathy, underpinning its beneficial effects on neurological complications in type 1 diabetes.

## 1.* *INTRODUCTION

Diabetes is an increasingly common metabolic disorder that affects the nervous system in a variety of ways. It impacts on the peripheral nervous system (PNS) in a progressive fashion resulting in diabetic polyneuropathies (DPNs), which as a group is the most common chronic diabetic complication [1]. It also affects the central nervous system (CNS) resulting in progressive cognitive impairment and is associated with an increased risk for the development of Alzheimer's disease [2, 3]. The mechanisms underlying these complications are several and are not necessarily the same in type 1 and type 2 diabetes [2, 4–6]. Historically, hyperglycemia, which is a common clinical attribute of both types of diabetes, has been regarded as the major underlying factor initiating the complications. However, this does not explain differences in the neurological complications in the two types of diabetes, nor does it explain the only partial benefits in curbing the progression or preventing the complications in trials aimed at optimal hyperglycemic control, such as the DCCT and UKPDS trials [7, 8]. Downstream effects of hyperglycemia on the polyol pathway and oxidative stress have been the targets for numerous clinical trials with marginal effects at best [9–11]. These data strongly suggest that factors other than hyperglycemia are involved in the initiation and progression of DPN. Such factors may differ in the two types of diabetes as suggested by epidemiological studies. The prevalence of DPN in type 2 diabetes is about 50% after 25 years of diabetes, whereas in type 1 diabetes it is close to 100% after 15-years disease duration [12–14], suggesting a more rapid progression of DPN in type 1 diabetic subjects.

DPN involves both somatic and autonomic peripheral nerves and is characterized as a progressive dying back axonopathy. The structural pathology, however, differs in the two types of diabetes in that the axonopathy is more severe in type 1 DPN and is in type 2 DPN associated with a greater frequency of primary segmental demyelination. Type 1 DPN is also characterized by progressive nodal and paranodal degeneration with significant impact on nerve function, abnormalities which do not occur in type 2 diabetes [4, 5, 15].

One factor that differs between type 1 and type 2 diabetes and is likely the explanation for some of the differences in DPN is the degree of perturbed insulin signaling due to insulin deficiency in type 1 diabetes and insulin resistance associated with hyperinsulinemia in type 2 diabetes. Insulin signaling exerts besides its hypoglycemic effect a multitude of metabolic and molecular effects, which are not commonly recognized. Pertaining to DPN, insulin signaling has prominent effects on Na^+^/K^+^-ATPase and NO activities important for the metabolically induced acute nerve dysfunction. It transduces strong neurotrophic effects on its own and possesses generegulatory functions on other neurotrophic factors such as IGF-1, NGF, and NT-3 as well as their receptors. Furthermore, it is an important regulator of postranslational modifications of neuroskeletal and cell adhesive proteins, and besides that it possesses a strong antiapoptotic effect. Considering these effects, it is not totally surprising that strict hyperglycemic control alone will not provide total protection against DPN [7, 8], or CNS for that matter, in diabetic subjects and that mechanistic, functional, and structural differences exist between the neurological complications occurring in the two types of diabetes [16].

Insulin is secreted from pancreatic beta cells in response to glucose. Simultaneously, proinsulin C-peptide is secreted in equimolar quantities. Insulin's half-life in the circulation is short whereas that of C-peptide is substantially longer [17, 18]. C-peptide was initially believed to be a waste product of insulin synthesis until the molecular bases for its intriguing insulin-like effects were delineated [19, 20].

In this review, we will briefly describe recent data pertaining to the interaction between insulin and C-peptide, outline the pathogenetic mechanisms underlying type 1 DPN, and contrast these to those of type 2 DPN. We will describe the effects of C-peptide on somatic and small fiber neuropathy and briefly summarize the effects on primary diabetic encephalopathy.

## 2. INSULIN AND C-PEPTIDE INTERACTIONS

After its discovery in the 1960s by Steiner [21–23], it was believed that C-peptide, which plays an intricate part in the biosynthesis and folding of insulin, would have an insulin-like glucose lowering effect. Since this turned out not to be the case, C-peptide was abandoned and dismissed as a nonfunctional peptide. However, in the 1990s, the Karolinska group and others demonstrated effects on blood flow, incipient diabetic nephropathy, and neuropathy in type 1 diabetic subjects [24–27]. This led to renewed interests in the action of C-peptide. The Karolinska group demonstrated specific binding of C-peptide to cell surfaces and suggested that it acted via a G-protein-related receptor mechanism [28]. Detailed studies by Grunberger et al. [19, 20, 29] demonstrated that C-peptide autophosphorylates the insulin receptor in the presence of insulin and stimulates p38 MAP-kinase and PI-3 kinase activity and reduces the activation of JNK phosphorylation, with subsequent dose-related effects on Na^+^/K^+^-ATPase activity and NO [30–32]. These experiments seemed to suggest an insulinomimetic effect, although despite years of effort by us and the Karolinska group, we failed to identify a specific C-peptide receptor. Further studies revealed an interesting stoichiometric relationship between insulin and C-peptide pertaining to insulin signaling activities. It was shown that in the presence of high concentrations of insulin, C-peptide has an inhibitory effect on the combined insulin-signaling activity, whereas in the presence of low insulin concentrations C-peptide enhances insulin signaling [19, 20, 33]. Recent data have suggested that the enhanced insulinomimetic effect displayed by C-peptide is due to its ability to dehexamerize insulin and thereby enhance the intrinsic actions of insulin itself [34]. As of yet unpublished data have demonstrated that the effects exerted by C-peptide on insulin action can be prolonged by its binding of metal-ions such as chromium and iron. It therefore appears that C-peptide interacts in a complex way with insulin to produce its supporting insulinomimetic effects.

## 3. MECHANISMS UNDERLYING TYPE 1 AND TYPE 2 DPN

**Figure 1:**
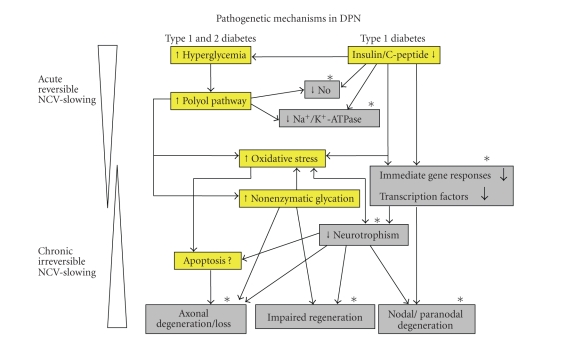
Scheme of pathogenetic events in type 1 (hyperglycemic and insulin and C-peptide deficient) and type 2 (hyperglycemic) BBZDR/Wor-rats. Insulin and C-peptide deficiencies add significantly to early metabolic abnormalities such as Na^+^/K^+^-ATPase and NO activities underlying the acute and reversible nerve conduction defect (dark gray). Subsequent changes with respect to gene regulatory mechanisms and suppression of major neurotrophic factors and their receptors lead to severe axonal degeneration, atrophy, and loss; nodal and paranodal degenerative changes; and impaired nerve fiber regeneration (dark gray). Such changes are responsible for the chronic and increasingly irreversible nerve dysfunction, which are more severely expressed in type 1 diabetes. Mechanisms on which C-peptide has preventive or corrective effects are indicated with (*).

The progressive evolution of pathogenetic factors responsible for DPN can be divided into an early and reversible metabolic phase and a partly overlapping progressively irreversible structural phase [1, 35] (Figure 1).

**Figure 2:**
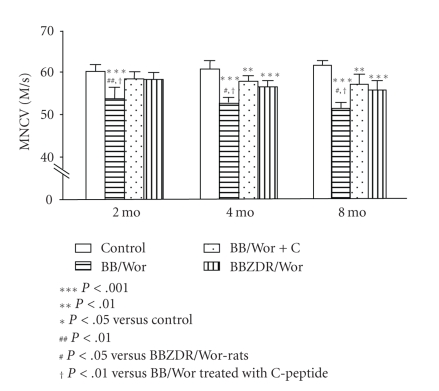
Longitudinal measurements of motor nerve conduction velocities (MNCVs) in the sciatic-tibial conducting system. Note a progressive decline in MNCV in type 1 BB/Wor-rats with duration of diabetes.This decline is significantly milder in type 2 BBZDR/Wor-rats and only become significant after 4 months of diabetes. C-peptide replacement from onset of diabetes had significant effects on MNCVs although these are not completely prevented [17, 39, 47].

An early metabolic perturbation is activation of the polyol pathway by excessive glucose, resulting in accumulation of sorbitol and fructose and depletion of other osmolytes such as taurin and myoinositol [36–38]. Myoinositol depletion results in insufficient diacylglycerol for Na^+^/K^+^-ATPase activation [36]. The more severe Na^+^/K^+^-ATPase defect in type 1 DPN is accounted for the additional defects in protein kinase C activity caused by insulin and C-peptide deficiencies [39] (Figure 1). Impaired endoneurial blood flow underlies endoneurial hypoxemia caused by impaired eNOS expression and NO activity, abnormalities, which are magnified by insulin and C-peptide deficiencies [32, 40, 41] (Figure 1). These aberrations have also been associated with hyperglycemia-induced mitochondrial dysfunction, overproduction of superoxide, oxidative, and nitrosative stress [41, 42]. Such early reversible metabolic abnormalities are associated with nerve conduction slowing, which is significantly more severe in type 1 BB/Wor-rats than in their type 2 counterparts the BBZDR-rats [39, 43] (Figure 2). These differences appear to be mainly due to differences in the Na^+^/K^+^-ATPase defect [37, 39, 43, 44]. Since the excitation of the nodal membrane underlying the propagation of nerve conduction depends on the inward flux of Na^+^, decreased Na^+^/K^+^-ATPase activity results in improper inactivation of intra-axonal Na^+^ with decreased permeability and intra-axonal Na^+^ accumulation, potentially resulting in conduction block [45, 46].

**Figure 3:**
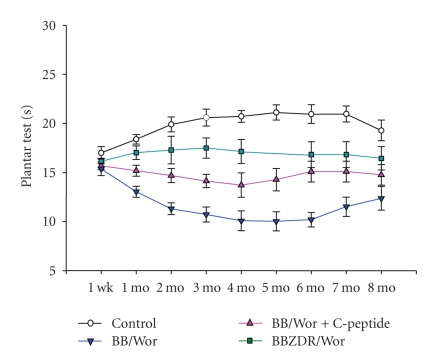
Longitudinal measurements of thermal hyperalgesia in type 1 BB/Wor-rats without and with C-peptide replacement from onset of diabetes compared to duration- and hyperglycemia-matched type 2 BBZDR/Wor-rats and age-matched control rats. Note more severe hyperalgesia in type 1 as compared to type 2 rats and with partial but significant prevention in C-peptide treated rats [48, 49].

Functional abnormalities of small nerve fibers, particularly unmyelinated fibers and small myelinated A*δ* fibers, occur early and underlie hyperalgesia and allodynia or neuropathic pain [48–50] (Figure 3). This is associated with increased formation of Na^+^-channels and *α*-adrenergic receptors resulting in hyperexcitability and ectopic discharges in C-fibers, which appears to be the initiating event [51–54]. Other mechanisms which contribute to and sustain pain are related to remodeling of large A*β* fibers which form collaterals with excitotoxic effects on nociceptive spinal cord neurons which amplify pain [54, 55]. Additional central nervous system mechanisms involving noradrenalin and serotonin reuptake as well as gabaergic effects are involved leading to different levels of sensitization of pain. These functional defects occur earlier and to a greater extent in type 1 DPN as compared to that of type 2 DPN [48] (Figure 3). On the other hand, hyperalgesia appears to persist for a longer period of time in type 2 diabetic rats (Figure 3), which may explain the fact that nociceptive neuropathy is more common in type 2 than in type 1 patients [56]. The early damage to small peripheral nerve fibers appears to result from decreased neurotrophic support by insulin and nerve growth factor (NGF) both of which are particularly neurotrophic to small nociceptive ganglion cells [57, 58].

Impaired insulin and/or NGF support may also explain the occurrence of painful diabetic neuropathy in prediabetic patients with impaired insulin function [59, 60], and nociceptive neuropathy in prediabetic rats with impaired glucose tolerance but without overt hyperglycemic diabetes [61]. It therefore appears that although hyperglycemia remains an important factor in the pathogenesis of DPN, differences in metabolic influences due to the presence or absence of insulin action modulate the severity of DPN and is likely to be the main explanation for the differences in DPN between the two types of diabetes.

**Figure 4:**
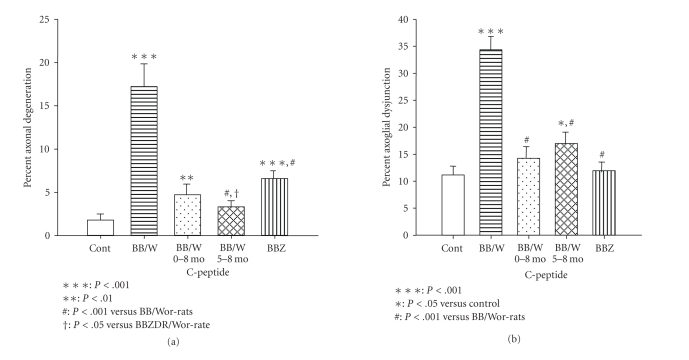
Magnitudes of myelinated axon degeneration as assessed by teased fiber analysis (a) and ultrastructural quantification of axoglial dysjunction (b), a measure of paranodal degeneration. Note a significantly more severe axonal degeneration in type 1 as compared to type 2 rats. C-peptide treatments from onset of diabetes and as an intervention between 5 and 8 months had significant preventive and corrective effects on axonal degeneration. In (b) type 2 diabetes was not affected by paranodal degeneration in contrast to type 1 diabetes showing profound degeneration. C-peptide treatments had significant preventive and therapeutic effects on paranodal degeneration [17, 39, 47].

**Figure 5:**
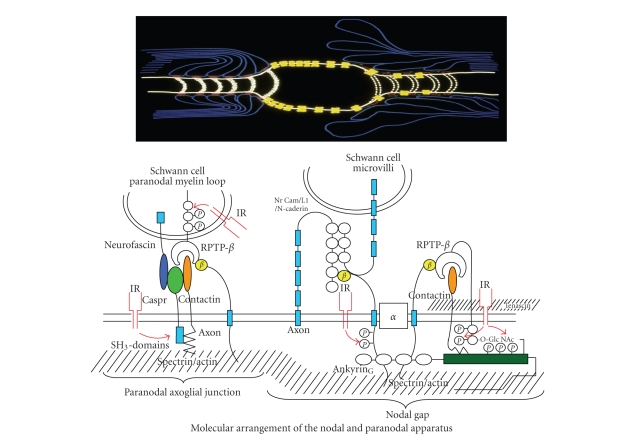
Schematic illustration of the nodal and paranodal molecular architecture in the normal situation (top left) and in the type 1 DPN (top right). The intricate relationships between several paranodal adhesive molecules emanating from the terminal myelin loops and the paranodal axolemma are depicted. Note the colocalization of the insulin receptor (IR) (bottom left). At the node the gated Na-*α*-channels are “anchored” to the axolemma via interaction with *β*-Na^+^-channels, RPTP*β*, contactin, and their interaction with ankyrin_G_, (bottom right). For further explanation of the molecular perturbations in type 1 diabetes and the effect of C-peptide, see text [18].

The structural and progressively irreversible DPN is characterized by axonal atrophy and loss, which is more severely expressed in type 1 as compared to type 2 DPN in experimental diabetes [39, 43, 48] (Figures 1 and 4). Additional changes that characterize experimental and human type 1 DPN is a progressive degenerative process affecting the paranodal and nodal apparati [4, 5, 16, 62] (Figure 5). On the other hand, primary segmental degeneration is a more common feature of type 2 human and experimental diabetes, which may relate to abnormalities in caveolin-1 signaling, which in turn is modulated by cholesterol levels [4, 5, 16, 39, 63].

Cytoskeletal neurofilaments (NFs) and tubulins are major constituents of the axon and their expression levels and phosphorylation status determine axonal function and size [64, 65]. Reduced expression of NFs and tubulins occurs in experimental models of diabetes [66–69] and is associated with decreased axonal transport of NFs [70, 71] due to aberrant phosphorylation by phosphorylating protein kinases [71–73]. NFs are unique to neurons and interact with microtubules thereby forming the basis for axonal transport. NFs consist of three intermediate filaments, NFL, NFM, and NFH, forming coiled-coil dimers which align in a staggered fashion. Several neurotrophic factors like NGF, NT-3, IGF-1, insulin, and C-peptide stabilize NF transcripts [14, 74, 75]. Aberrant phosphorylation of NFs perturbs their function and interaction with other cytoskeletal components resulting in malalignment of the cytoskeleton, impaired axonal function, atrophy, and eventually loss [14, 76–78]. Several kinases are involved in NF phosphorylation such as cyclin-dependent kinases including Cdk5 and the MAP kinases Erk 1/2, SAPK [72], and GSK*β* [79–81].

**Figure 6:**
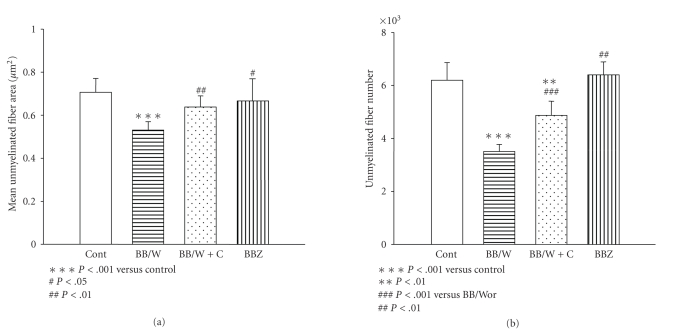
The effect of type 1 and type 2 diabetes on unmyelinated axonal size (a) and numbers (b) in the sural nerve in 7-8 month diabetic rats. Note in type 1 rats, significant atrophy (a) and loss (b) of unmyelinated fiber, whereas no significant deficits were detectable in type 2 BBZDR/Wor-rats. Replenishment with C-peptide resulted in significant prevention of C-fiber atrophy (a) and loss (b) [17, 47].

Tubulins assemble into microtubules and provide for axonal transport and polarity [1]. Microtubule-associated proteins like MAP1B and tau regulate their assembly [82–84]. Inhibition of GSK-3*β* abolishes MAP1B phosphorylation which impacts on microtubule stability [85]. Reduced expression of NFs and tubulins occur already in 2-mo diabetic type 1 BB/Wor-rats and tend to progress with duration of diabetes, whereas similar changes occur later and are significantly milder in type 2 BBZDR/Wor-rats [43, 66]. Simultaneously, neurofilaments become hyperphosphorylated in type 1 diabetic rats via upregulation of phosphorylating stress kinases like SAPK and GSK-3*β* which emanate from impaired insulin, IGF-1, and C-peptide signaling [86]. The structural consequences as would be expected, therefore affect type 1 DPN more severely than type 2 DPN with unmyelinated fibers being particularly vulnerable [16, 48] (Figures 2, 3, 4, and 6). A further difference between type 1 and type 2 DPN occurs in sympathetic autonomic nerves. STZ- and BB/Wor-rats develop dystrophic axonal changes consisting of accumulations of NFs, tubolovesicular conglomerates, and degenerated organelles. These changes have been related to insulin and IGF-1 deficits and do not occur to a significant degree in type 2 BBZDR/Wor-rats [87].

The differences in insulin-deficiency-mediated effects on neurotrophic factors and downstream deficits in the expression and phosphorylation status of neuroskeletal proteins also affect the regenerative capacity of injured nerves. Hence, the immediate gene responses following nerve injury and upregulation of the expression of neuroskeletal, mRNAs, and proteins are more severely perturbed in type 1 BB/Wor-rats as compared to their type 2 counterpart, the BBZDR/Wor-rats [43, 66, 69].

In recent years, it has been suggested by several investigators [88, 89] that DPN is in part caused by mitochondrial dysfunction-related apoptosis of dorsal root ganglion cells. However, it is difficult to reconcile this loss of DRG neurons in the absence of peripheral sensory nerve fiber loss in the streptozotocin diabetic rat. Although apoptotic stresses do occur, more so in type 1 diabetic DRG cells than in those of type 2 diabetes, these appear to be counteracted by antiapoptotic mechanism [90, 91]. Instead the degeneration and eventually loss, particularly of small nociceptive neurons, of DRGs in type 1 BB/Wor-rats appear to be due to degeneration and vacuolation of the Golgi apparatus [92].

Probably the most intriguing difference encountered in DPN in the two types of diabetes is the progressive degeneration of the paranodal ion-channel barrier in type 1 DPN, which is unaffected in DPN accompanying type 2 diabetes [4, 5, 39, 62] (Figures 1 and 5). This abnormality when first described [4, 62] caused some controversy, since it could not be identified in mostly type 2 diabetic nerve [93–95]. The tight junctions which make up the paranodal barrier are composed of cell adhesive molecules localized to the axolemma such as casper, Na *β*-channels and contactin and receptor protein tyrosin phosphatase *β* (RPTP-*β*) on the terminal loops of the myelin sheath (Figure 5). The interaction of these adhesive molecules depends on their posttranslational modifications, which become progressively compromised in type 1 DPN, resulting in a breakup of tight junctional structures and the barrier itself [62, 96–99]. Simultaneous defects in Na *β*-channels and ankyrin_G_ of the nodal axolemma dislodge the Na-*α*-channels which become lateralized [97–99] (Figure 5). These abnormalities result in decreased density of nodal Na-*α*-channels with profound consequences as to the propagation of conduction impulses [45, 62, 96–98]. Interestingly, the insulin receptor, which is markedly downregulated in type 1 diabetes, colocalizes with paranodal tight junctions and decorates the nodal axolemma [100].

## 4. THE EFFECT OF C-PEPTIDE REPLACEMENT ON TYPE 1 DPN

Initial in vitro studies on the effect of C-peptide, demonstrated insulin-like effects [19, 20, 29, 101–105]. With regard to DPN, we and several other groups demonstrated a dose-related beneficial effect on neural Na^+^/K^+^-ATPase activity [17, 31, 47], which constitutes the most important early metabolic abnormality with consequences pertaining to nerve conduction velocity as outlined above. Neurovascular dysfunction associated with oxidative stress has emerged as a contributing factor in the acute development of DPN [42, 106–109].

C-peptide promotes the release of NO in endothelial cells in a concentration-dependent manner [110]. In addition, it increases the expression of eNOS protein and mRNA which appears to be mediated via a MAP-kinase-dependent mechanism [102, 110–112]. These observations are consistent with in vivo findings in humans and animal models [24, 25, 27, 32, 33, 113, 114].

The effect of C-peptide replacement in type 1 BB/Wor-rats, resulted in correction of endoneurial perfusion, the nerve conduction defect, and attenuated thermal hyperalgesia [32]. It did not demonstrate an effect on oxidative stress. Inhibition of eNOS, but not of cyclooxygenase, reversed the positive effects of C-peptide [32]. Interestingly, in hyperglycemia-matched type 2 BBZDR/Wor-rats, neurovascular deficits and increased oxidative stress were not accompanied by nerve conduction slowing or hyperalgesia [32]. These findings indicate that sensory nerve conduction deficits and small fiber function are not inevitably consequences of increased oxidative stress or decreased endoneurial blood flow in this type 2 rodent model [32].

**Figure 7:**
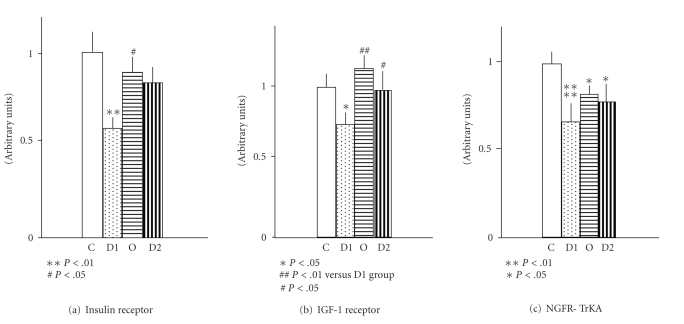
Expression of the receptors of neurotrophic factors in dorsal root ganglia in 8-month type 1 diabetic (D1) and type 2 diabetic (D2) rats, as well as type 1 rats replaced with C-peptide. (c) compared to age-matched control rats . Note marked decreases in the expression of insulin receptor, IGF-1 receptor, and NGF-TrkA receptor in type 1 rats. These defects were significantly milder in type 2 diabetic rats and were significantly prevented by C-peptide replacement in type 1 diabetic rats [50].

Insulin and C-peptide exert on their own neurotrophic and antiapoptotic effects [115–117]. In addition, C-peptide has corrective effects on the expression of several neurotrophic factors such as NGF, IGF-1, and NT-3 and their respective receptors [49, 50, 118] (Figure 7). These regulatory effects appear to be mediated by early gene regulatory effects of c-fos particularly on NGF as well as by transcriptional factor NF*κ*B with wider implications [66, 115]. The insulin receptor itself is in peripheral nerve located primarily to the paranodal and nodal regions of myelinated fibers and to small nociceptive neurons in the DRGs [58, 100].

In the sciatic nerve, the expression of the insulin receptor is upregulated in the BB/Wor-rat, whereas its expression in type 2 BBZDR/Wor-rats is downregulated by more than 50% [66], in contrast the insulin receptor expression becomes progressively downregulated in DRGs of the type 1 model and remains unchanged in type 2 rats [48]. Systemic IGF-1 is decreased in both models [3], whereas NGF and NF-3 are impaired in sciatic nerves of the BB/Wor-rat but not in the type 2 BBZDR/Wor-rat [48] and their respective receptors are significantly more severely affected in the type 1 model [48]. These aberrations in the expression of neurotrophic factors and their receptors in the BB/Wor-rat are fully prevented by full continuous substitution of C-peptide [49] and are significantly improved following intervention with C-peptide [50]. Such beneficial effects on the neurotrophic supporting network transcend into effects on major neuroskeletal proteins such as NFs and neurotubulins [86, 118], their postranslational modifications, and ultimately axonal size, a major determinant of axonal function, hence resulting in prevention and even reversal of nerve dysfunction [6, 17, 31, 49, 50] (Figures 2 and 3). As mentioned earlier, nociceptive DRG neurons are specifically responsive to insulin and NGF. It is therefore not totally surprising that nociceptive nerve fibers are particularly vulnerable to the diabetic insult. In the type 1 model, they are more severely affected than in the type 2 rat [48] showing a progressive axonal atrophy coupled with nociceptive neuronal atrophy with ultimate C-fiber loss and loss of substance P and calcitonin-gene-related neurons [49, 50]. The progressive distal fiber loss and subsequent neuronal atrophy and loss are not likely to reflect apoptotic cell death. Instead, apoptotic stresses which indeed do occur are likely to be counteracted by antiapoptotic elements such as heat shock proteins [119]. In a recent study, we demonstrated profound changes of the Golgi apparatus particularly in small sensory DRG neurons in the type 1 BB/Wor-rat and suggested that this may reflect neurotrophic withdrawal with degeneration of cytoskeletal binding proteins and microtubules [92].

The impact of insulin-signaling on regulation of neurotrophic support is also reflected by the effect of C-peptide on normalizing nerve fiber regeneration in the BB/Wor-rat [118].

As mentioned above, one of the most characteristic abnormalities occurring in type 1 human and experimental diabetes is the progressive nodal and paranodal degeneration [4, 16, 62]. Axoglial dysjunction is a progressive degeneration of the paranodal ion-channel barrier which eventually results in paranodal degeneration and reparative intercalated internodes [4, 62]. This abnormality is not specific for type 1 DPN, but occurs in a series of clinical and experimental neuropathies [120]. At the node, the voltage-gated Na^+^ *α*-channels are held in place by auxiliary subunits *β*_1_ and *β*_2_ Na-channels which act as adhesive molecules. Interaction between contactin, ankyrin_G_, and *β*-subunits are critical for the enrichment and localization of Na^+^ *α*-channels to the nodal axolemma. Ankyrin_G1_ interacts with other nodal cell adhesion molecules and its postranslational modifications are important for these interactions. It interacts with the Na-channel *β*-subunits which in turn interact with RPTP-*β*.

At the paranode, the myelin loops adhere to the axolemma via tight junctions. Caspr is part of these and interacts with contactin and RPTP-*β*. Caspr's cytoplasmic tail mediates protein-protein interaction through binding with p85 at SH_3_ domains [99]. In myelinated nerve fibers, insulin receptors are particularly concentrated to the node and the paranode [100]. In type 1 DPN, caspr and contactin become significantly downregulated together with RPTP-*β* associated with a defect in caspr's p85 binding. p85, the regulatory subunit of phosphatidyl-inositol 3-kinase, is possibly mediated by insulin signaling (Figure 5). This sequence of events leads to disruption of the tight junctions [99]. At the node of Ranvier, the expression of Na^+^-channel *α*-subunits is not altered, although the *β*_1_-subunit is downregulated together with contactin and ankyrin_G_. In addition, the latter undergoes O-linked N-acetylglucosylation, which inhibits its phosphorylation and interaction with the Na-channel *β*-units and contactin. This leads to dislodgement of Na-channel *α*-subunits, which now migrate laterally through the breached paranodal ion-channel barrier [97, 99].

C-peptide substitution in type 1 BB/Wor-rats prevents the degenerative processes of the paranode and the node of Ranvier [99] and intervention with C-peptide repairs the paranodal apparatus as evidenced by an increased number of intercalated internodes [17]. It therefore appears as if these functionally significant lesions in type 1 DPN relate to abnormalities in insulin-signaling.

## 5. PRIMARY DIABETIC ENCEPHALOPATHY IN TYPE 1 DIABETES AND THE EFFECT OF C-PEPTIDE

Cognitive deficits occur more commonly in diabetic patients than in the nondiabetic population [121–125]. This is probably in part due to ischemic pathologies due to cerebral micro- and macrovascular disease, which may be confounded by hypertensive cerebral angiopathy or to repeated episodes of severe hypoglycemia. Such conditions have been referred to as secondary diabetic encephalopathy. However, there is now growing evidence to suggest that cognitive impairments may be consequent to perturbed metabolism in diabetes or so-called primary diabetic encephalopathy [126]. Impaired memory, problem solving ability, and intellectual development have been documented in patients with type 1 diabetes. Such signs and symptoms have been accompanied by electrophysiological and structural abnormalities [127–130]. These appear to be more common in patients with early onset of diabetes and may in part relate to interference with normal brain development [124, 131, 132].

Cognitive decline in patients with type 2 diabetes may be associated with an increased risk for the development of Alzheimer's disease due to CNS insulin resistance and other confounding factors, such as overweight and hypercholesterolemia [2, 122, 123].

Deficits in cognitive function have also been documented in experimental models of diabetes. In the streptozotocin-induced diabetic rat, impaired cognitive performances have been associated with abnormalities in hippocampal long-term potentiation indicative of abnormal synaptic plasticity, changes that are reversed by insulin treatment [133, 134]. We have demonstrated that impaired spatial memory in diabetic BB/Wor-rats is preceded by significant reductions in the expression of IGF-1, IGF-II, IGF-1 receptor and insulin receptor in hippocampus in 2 months diabetic rats [135]. These early findings were followed by increasingly impaired deficits in Morris water maze-testing, laddering of genomic DNA in hippocampus and frontal cortex associated with elevated Bax/Bcl-X_*L*_ ratios, increased caspase 3 activity, and neuronal loss in hippocampus [117, 135]. In these studies, full replacement with proinsulin C-peptide attenuated the functional cognitive deficits, normalized hippocampal expression of insulin and IGF-1 receptors, Bax expression, and that of cleaved PARP, active caspase 3, and caspase 12. These effects were associated with significant reductions in hippocampal neuronal loss [117, 136].

On the other hand, in a recent study [3] of the type 2 BBZDR/Wor-rat, we demonstrated in the frontal cortex perturbed amyloid precursor protein (APP) metabolism with increased accumulation of *β*-amyloid, soluble APP, and a 3-fold increase of A*β* C-terminal fragments. These changes were associated with insulin resistance and decreased expression of insulin and IGF receptors and increased deposition of phospho-tau. The consequence of these abnormalities was decreased synapse density, neuritic degeneration, and neuronal loss [2, 3]. Parallel studies in the type 1 counterpart, the BB/Wor-rat, showed similar changes although they were significantly milder as compared to type 2 rats [3]. Interestingly though, amyloid deposition and increased phospho-tau were not affected by C-peptide replacement (unpublished data, Li and Sima).

It is therefore clear that cognitive deficits occur in rodent models of diabetes, which have not been genetically manipulated. The underlying molecular abnormalities appear to differ in type 1 and type 2 diabetes. In the former, it appears to be mainly caused by a deficit in insulin signaling and availability of neurotrophic support, which can be modified by C-peptide replacement. In contrast, the rather profound Alzheimer-like changes in type 2 diabetes appear to relate to insulin-resistance and possibly elevated cholesterol levels, abnormalities which do not appear to be responsive to C-peptide treatment.

## 6. CONCLUDING THOUGHTS AND APPEALS

It is becoming increasingly evident that DPN differs in the two types of diabetes. This is not totally surprising when considering the underlying pathophysiologic differences between type 1 and type 2 diabetes. The only commonality of the two disorders is hyperglycemia. Although hyperglycemia remains a prominent factor in the pathogenesis of the chronic complications, probably equally important is the role of insulin or lack thereof together with its prime assistant C-peptide. Recognizing such differences will open up areas of untapped therapeutic possibilities. One of these concerns C-peptide. As outlined in this review, unlike earlier examined therapeutic approaches which have met with disappointing results, C-peptide corrects a number of key pathogenetic mechanisms involved in DPN and has experimentally and in limited clinical trials proven to be highly efficacious in preventing and even reversing DPN in type 1 diabetes. In view of this, it is surprising that major insulin manufacturing companies as well as main granting agencies have approached this new evolving area with such skepticism. The overriding concept is almost embarrassingly simple: since the discovery of insulin and the lack thereof in type 1 diabetes, we have for more than 80 years replaced it in type 1 patients and thereby saved millions of lives, who however still develop the late complications with significant disabilities. Would not it now be about time to also replace insulin's companion and thereby prevent millions of type 1 patients from developing the devastating late complications? This concept takes on an even greater dimension and urgency, when considering the preliminary data eluded to in this review, indicating the potential effect of C-peptide substitution in preventing cognitive impairments and even dementia in type 1 diabetic patients. Therefore, we appeal to the pharmaceutical industry and federal and private agencies to get involved. A great leap in the treatment of type 1 diabetes may be just around the corner.

## Figures and Tables

**Table 1:** Summary of the corrective effects of C-peptide on metabolic, molecular, functional, and structural parameters in DPN and primary diabetic encephalopathy. Arrows indicate a decrease (↓) or increase (↑) in the parameter in the non-C-peptide-treated situation. The original findings are referenced.

Diabetic Neuropathy	
Abnormality	References
↓Na^+^/K^+^-ATPase	[29, 88]
↓eNOS, NO	[31, 95]
↓c-fos	[63]
↓NF*κ*B	[84]
↓NGF, TrkA	[49, 50, 98]
↓IGF-1, IGF-1R	[49, 50, 98]
↓NT-3, TrkC	[49, 50]
↓Insulin Receptor	[49, 50]
↓Neurofilaments	[74, 98]
↓Tubulins	[74, 98]
↓Substance P	[49, 50]
↓CGRP	[49, 50]
↓Cell adhesive molecules	[82]
↑DRG neuronal atrophy	[49, 50]
and loss	
↓NCV	[16, 29, 31, 49, 50, 74, 82, 88, 95]
↑Axonal atrophy and loss	[16, 49, 50, 74, 88, 98]
↓Nerve fiber regeneration	[98]
↑Axoglial dysjuction	[16, 82, 88]
*Primary Diabetic Encephalopathy*	
↓Insulin Receptor	[96, 97, 104, 114, 115]
↓IGF-1, IGF-1R	[97, 104, 114, 115]
↑Caspase 3	[114, 115]
↑Caspase 12	[114, 115]
↑Tau	Unpublished data
↑Apoptosis	[113, 115]
Cognitive function	[114, 115]

## References

[1] Sima AAF (2001). Diabetic neuropathy: pathogenetic background, current and future therapies. *Expert Review of Neurotherapeutics*.

[2] Sima AAF, Li Z-G Diabetes, cognitive dysfunction and Alzheimer's disease.

[3] Li Z-G, Zhang W, Sima AAF (2007). Alzheimer-like changes in rat models of spontaneous diabetes. *Diabetes*.

[4] Sima AAF, Nathaniel V, Bril V, McEwen TAJ, Greene DA (1988). Histopathological heterogeneity of neuropathy in insulin-dependent and non-insulin-dependent diabetes, and demonstration of axo-glial dysjunction in human diabetic neuropathy. *Journal of Clinical Investigation*.

[5] Sima AAF (2004). Diabetes underlies common neurological disorders. *Annals of Neurology*.

[6] Sima AAF (2004). Diabetic neuropathy in type 1 and type 2 diabetes and the effects of C-peptide. *Journal of the Neurological Sciences*.

[7] The Diabetes Control and Complications Trial Research Group (1993). The effect of intensive treatment of diabetes on the development and progression of long-term complications in insulin-dependent diabetes mellitus. *The New England Journal of Medicine*.

[8] UK Prospective Diabetes Study (UKPDS) Group (1998). Intensive blood glucose control with sulphonylureas or insulin compared with conventional treatment and risk of complications in patients with type 2 diabetes (UKPDS 33). *The Lancet*.

[9] Sima AAF, Bril V, Nathaniel V (1988). Regeneration and repair of myelinated fibers in sural-nerve biopsy specimens from patients with diabetic neuropathy treated with sorbinil. *The New England Journal of Medicine*.

[10] Pfeifer MA, Schumer MP, Gelber DA (1997). Aldose reductase inhibitors: the end of an era or the need for different trial designs?. *Diabetes*.

[11] Ziegler D, Hanefeld M, Ruhnau K-J (1999). Treatment of symptomatic diabetic polyneuropathy with the antioxidant *α*-lipoic acid: a 7-month multicenter randomized controlled trial (ALADIN III study). *Diabetes Care*.

[12] Vinik AI, Holland MT, Le Beau JM, Liuzzi FJ, Stansberry KB, Colen LB (1992). Diabetic neuropathies. *Diabetes Care*.

[13] Tesfaye S, Stevens LK, Stephenson JM (1996). Prevalence of diabetic peripheral neuropathy and its relation to glycaemic control and potential risk factors: the EURODIAB IDDM complications study. *Diabetologia*.

[14] Sima AAF (2003). New insights into the metabolic and molecular basis for diabetic neuropathy. *Cellular and Molecular Life Sciences*.

[15] Sima AAF (2003). C-peptide and diabetic neuropathy. *Expert Opinion on Investigational Drugs*.

[16] Sima AAF, Kamiya H, Adeghate E, Saadi H, Adem A, Obineche E (2006). Diabetic neuropathy differs in type 1 and type 2 diabetes. *Diabetes Mellitus and Its Complications. Molecular Mechanisms, Epidemiology and Clinical Medicine*.

[17] Zhang W, Kamiya H, Ekberg K, Wahren J, Sima AAF (2007). C-peptide improves chronic diabetic neuropathy in type 1 diabetic BB/Wor-rats. *Diabetes/Metabolism Research and Reviews*.

[18] Sima AAF, Kamiya H (2004). Insulin, C-peptide and diabetic neuropathy. *Science & Medicine*.

[19] Grunberger G, Qiang X, Li Z-G (2001). Molecular basis for the insulinomimetic effects of C-peptide. *Diabetologia*.

[20] Grunberger G, Sima AAF (2004). The C-peptide signaling. *Experimental Diabesity Research*.

[21] Steiner DF, Cunningham D, Spigelman L, Aten B (1967). Insulin biosynthesis: evidence for a precursor. *Science*.

[22] Steiner DF, Rubenstein AH (1997). Proinsulin C-peptide-biological activity?. *Science*.

[23] Kitabchi AE (1977). Proinsulin and C peptide: a review. *Metabolism*.

[24] Fernqvist-Forbes E, Johansson B-L, Eriksson MJ (2001). Effects of C-peptide on forearm blood flow and brachial artery dilatation in patients with type 1 diabetes mellitus. *Acta Physiologica Scandinavica*.

[25] Johansson B-L, Linde B, Wahren J (1992). Effects of C-peptide on blood flow, capillary diffusion capacity and glucose utilization in the exercising forearm of type 1 (insulin-dependent) diabetic patients. *Diabetologia*.

[26] Johansson B-L, Borg K, Fernqvist-Forbes E, Kernell A, Odergren T, Wahren J (2000). Beneficial effects of C-peptide on incipient nephropathy and neuropathy in patients with type 1 diabetes mellitus. *Diabetic Medicine*.

[27] Forst T, Kunt T, Pohlmann T (1998). Biological activity of C-peptide on the skin microcirculation in patients with insulin-dependent diabetes mellitus. *Journal of Clinical Investigation*.

[28] Rigler R, Pramanik A, Jonasson P (1999). Specific binding of proinsulin C-peptide to human cell membranes. *Proceedings of the National Academy of Sciences of the United States of America*.

[29] Li Z-G, Qiang X, Sima AAF, Grunberger G (2001). C-peptide attenuates protein tyrosine phosphatase activity and enhances glycogen synthesis in L6 myoblasts. *Biochemical and Biophysical Research Communications*.

[30] Ido Y, Vindigni A, Chang K (1997). Prevention of vascular and neural dysfunction in diabetic rats by C- peptide. *Science*.

[31] Zhang W, Yorek M, Pierson CR, Murakawa Y, Breidenbach A, Sima AAF (2001). Human C-peptide dose dependently prevents early neuropathy in the BB/Wor-rat. *International Journal of Experimental Diabetes Research*.

[32] Stevens MJ, Zhang W, Li F, Sima AAF (2004). C-peptide corrects endoneurial blood flow but not oxidative stress in type 1 BB/Wor-rats. *American Journal of Physiology*.

[33] Jensen ME, Messina EJ (1999). C-peptide induces a concentration-dependent dilation of skeletal muscle arterioles only in presence of insulin. *American Journal of Physiology*.

[34] Shafqat J, Melles E, Sigmundsson K (2006). Proinsulin C-peptide elicits disaggregation of insulin resulting in enhanced physiological insulin effects. *Cellular and Molecular Life Sciences*.

[35] Sima AAF (2007). Acetyl-L-carnitine in diabetic polyneuropathy: experimental and clinical data. *CNS Drugs*.

[36] Greene DA, Lattimer SA, Sima AAF (1987). Sorbitol, phosphoinositides, and sodium-potassium-ATPase in the pathogenesis of diabetic complications. *The New England Journal of Medicine*.

[37] Stevens MJ, Feldman EL, Thomas TP, Greene DA, Veves A, Corn PMC (1997). The pathogenesis of diabetic neuropathy. *Clinical Management of Diabetic Neuropathy*.

[38] Dvornik D, Porte D (1987). Hyperglycemia in the pathogenesis of diabetic complications. *Aldose Reductase Inhibition*.

[39] Sima AAF, Zhang W, Xu G, Sugimoto K, Guberski D, Yorek MA (2000). A comparison of diabetic polyneuropathy in type 2 diabetic BBZDR/Wor-rats
and in type 1 diabetic BB/Wor-rats. *Diabetologia*.

[40] Cameron NE, Cotter MA, Basso M, Hohman TC (1997). Comparison of the effects of inhibitors of aldose reductase and sorbitol dehydrogenase on neurovascular function, nerve conduction and tissue polyol pathway metabolites in streptozotocin-diabetic rats. *Diabetologia*.

[41] Wahren J, Ekberg K, Jörnvall H (2007). C-peptide is a bioactive peptide. *Diabetologia*.

[42] Brownlee M (2001). Biochemistry and molecular cell biology of diabetic complications. *Nature*.

[43] Pierson CR, Zhang W, Murakawa Y, Sima AAF (2003). Insulin deficiency rather than hyperglycemia accounts for impaired neurotrophic responses and nerve fiber regeneration in type 1 diabetic neuropathy. *Journal of Neuropathology & Experimental Neurology*.

[44] Sima AAF, Sugimoto K (1999). Experimental diabetic neuropathy: an update. *Diabetologia*.

[45] Brismar T, Sima AAF (1981). Changes in nodal function in nerve fibres of the spontaneously diabetic BB-Wistar rat: potential clamp analysis. *Acta Physiologica Scandinavica*.

[46] Sima AAF, Brismar T (1985). Reversible diabetic nerve dysfunction: structural correlates to electrophysiological abnormalities. *Annals of Neurology*.

[47] Sima AAF, Zhang W, Sugimoto K (2001). C-peptide prevents and improves chronic type I diabetic polyneuropathy in the
BB/Wor-rats. *Diabetologia*.

[48] Kamiya H, Murakawa Y, Zhang W, Sima AAF (2005). Unmyelinated fiber sensory neuropathy differs in type 1 and type 2 diabetes. *Diabetes/Metabolism Research and Reviews*.

[49] Kamiya H, Zhang W, Sima AAF (2004). C-peptide prevents nociceptive sensory neuropathy in type 1 diabetes. *Annals of Neurology*.

[50] Kamiya H, Zhang W, Ekberg K, Wahren J, Sima AAF (2006). C-peptide reverses nociceptive neuropathy in type 1 diabetes. *Diabetes*.

[51] Hirade M, Yasuda H, Omatsu-Kanbe M, Kikkawa R, Kitasato H (1999). Tetrodotoxin-resistant sodium channels of dorsal root ganglion neurons are readily activated in diabetic rats. *Neuroscience*.

[52] Lee Y-H, Ryu T-G, Park S-J (2000). *α*1-adrenoceptors involvement in painful diabetic neuropathy: a role in allodynia. *NeuroReport*.

[53] Burchiel KJ, Russell LC, Lee RP, Sima AAF (1985). Spontaneous activity of primary afferent neurons in diabetic BB/Wistar rats: a possible mechanism of chronic diabetic neuropathic pain. *Diabetes*.

[54] Zhang W, Murakawa Y, Wozniak KM, Slusher B, Sima AAF (2002). GCPII (NAALADase) inhibition prevents long-term diabetic neuropathy in type 1 diabetic
BB/Wor-rats. *Journal of the Neurological Sciences*.

[55] Woolf CJ, Shortland P, Reynolds M, Ridings J, Doubell T, Coggeshall RE (1995). Reorganization of central terminals of myelinated primary afferents in the rat dorsal horn following peripheral axotomy. *Journal of Comparative Neurology*.

[56] Quattrini C, Tesfaye S (2003). Understanding the impact of painful diabetic neuropathy. *Diabetes/Metabolism Research and Reviews*.

[57] Tomlinson DR, Fernyhough P, Sima AAF (2000). Neurotrophism in diabetic neuropathy. *Chronic Complications in Diabetes*.

[58] Sugimoto K, Murakawa Y, Sima AAF (2002). Expression and localization of insulin receptor in rat dorsal root ganglion and spinal cord. *Journal of the Peripheral Nervous System*.

[59] Singleton JR, Smith AG, Bromberg MB (2001). Increased prevalence of impaired glucose tolerance in patients with painful sensory neuropathy. *Diabetes Care*.

[60] Novella SP, Inzucchi SE, Goldstein JM (2001). The frequency of undiagnosed diabetes and impaired glucose tolerance in patients with idiopathic sensory neuropathy. *Muscle and Nerve*.

[61] Murakawa Y, Zhang W, Pierson CR (2002). Impaired glucose tolerance and insulinopenia in the GK-rat causes peripheral neuropathy. *Diabetes/Metabolism Research and Reviews*.

[62] Sima AAF, Lattimer SA, Yagihashi S, Greene DA (1986). Axo-glial dysjunction. A novel structural lesion that accounts for poorly reversible slowing of nerve conduction in the spontaneously diabetic bio-breeding rat. *Journal of Clinical Investigation*.

[63] Yu C, Ronen S, Dobrowsky R Downregulation of caveolin-1 in Schwann cells enhances ERBB2 activation and neuregulin-induced demyelination.

[64] Hoffman PN, Cleveland DW, Griffin JW, Landes PW, Cowan NJ, Price DL (1987). Neurofilament gene expression: a major determinant of axonal caliber. *Proceedings of the National Academy of Sciences of the United States of America*.

[65] de Waegh SM, Lee VM-Y, Brady ST (1992). Local modulation of neurofilament phosphorylation, axonal caliber, and slow axonal transport by myelinating Schwann cells. *Cell*.

[66] Pierson CR, Zhang W, Murakawa Y, Sima AAF (2002). Early gene responses of trophic factors in nerve regeneration differ in experimental type 1 and type 2 diabetic polyneuropathies. *Journal of Neuropathology & Experimental Neurology*.

[67] Mohiuddin L, Fernyhough P, Tomlinson DR (1995). Reduced levels of mRNA encoding endoskeletal and growth-associated proteins in sensory ganglia in experimental diabetes. *Diabetes*.

[68] Scott JN, Clark AW, Zochodne DW (1999). Neurofilament and tubulin gene expression in progressive experimental diabetes. Failure of synthesis and export by sensory neurons. *Brain*.

[69] Xu G, Murakawa Y, Pierson CR, Sima AAF (2002). Altered tubulin and neurofilament expression and impaired axonal growth in diabetic nerve 
regeneration. *Journal of Neuropathology & Experimental Neurology*.

[70] Medori R, Jenich H, Autilio-Gambetti L, Gambetti P (1988). Experimental diabetic neuropathy: similar changes of slow axonal transport and axonal size in different animal models. *Journal of Neuroscience*.

[71] Sayers NM, Beswick LJ, Middlemas A (2003). Neurotrophin-3 prevents the proximal accumulation of neurofilament proteins in sensory neurons of streptozotocin-induced diabetic rats. *Diabetes*.

[72] Terada M, Yasuda H, Kikkawa R (1998). Delayed Wallerian degeneration and increased neurofilament phosphorylation in sciatic nerves of rats with streptozotocin-induced diabetes. *Journal of the Neurological Sciences*.

[73] Fernyhough P, Gallagher A, Averill SA (1999). Aberrant neurofilament phosphorylation in sensory neurons of rats with diabetic
neuropathy. *Diabetes*.

[74] Middlemas A, Delcroix J-D, Sayers NM, Tomlinson DR, Fernyhough P (2003). Enhanced activation of axonally transported stress-activated protein kinases in peripheral nerve in diabetic neuropathy is prevented by neurotrophin-3. *Brain*.

[75] Lee MK, Xu Z, Wong PC, Cleveland DW (1993). Neurofilaments are obligate heteropolymers in vivo. *Journal of Cell Biology*.

[76] Grant P, Pant HC (2000). Neurofilament protein synthesis and phosphorylation. *Journal of Neurocytology*.

[77] Sima AAF (1983). The development and structural characterization of the neuropathies in the spontaneously diabetic BB Wistar rat. *Metabolism*.

[78] Sima AAF, Bouchier M, Christensen H (1983). Axonal atrophy in sensory nerves of the diabetic BB-Wistar rat: a possible early correlate of human diabetic neuropathy. *Annals of Neurology*.

[79] Purves T, Middlemas A, Agthong S (2001). A role for mitogen-activated protein kinases in the etiology of diabetic neuropathy. *The FASEB Journal*.

[80] Brownlees J, Yates A, Bajaj NP (2000). Phosphorylation of neurofilament heavy chain side-arms by stress activated protein
kinase-1b/Jun N-terminal kinase-3. *Journal of Cell Science*.

[81] Li BS, Veeranna, Gu J, Grant P, Pant HC (1999). Activation of mitogen-activated protein kinases (Erk 1 and Erk 2) cascade results in phosphorylation of NF-M tail domains in transfected NIH 3T3 cells. *European Journal of Biochemistry*.

[82] Mandelkow E, Mandelkow E-M (1995). Microtubules and microtubule-associated proteins. *Current Opinion in Cell Biology*.

[83] Nunez J, Fischer I (1997). Microtubule-associated proteins (MAPs) in the peripheral nervous system during development and regeneration. *Journal of Molecular Neuroscience*.

[84] Butner KA, Kirschner MW (1991). Tau protein binds to microtubules through a flexible array of distributed weak sites. *Journal of Cell Biology*.

[85] Zhou F-Q, Snider WD (2005). GSK-3 and microtubule assembly in axons. *Science*.

[86] Kamiya H, Zhang W, Sima AAF Progressive axonal degeneration in type 1 BB/Wor-rats.

[87] Schmidt RE, Dorsey DA, Beaudet LN, Parvin CA, Zhang W, Sima AAF (2004). Experimental rat models of types 1 and 2 diabetes differ in sympathetic
neuroaxonal dystrophy. *Journal of Neuropathology & Experimental Neurology*.

[88] Russell JW, Sullivan KA, Windebank AJ, Herrmann DN, Feldman EL (1999). Neurons undergo apoptosis in animal and cell culture models of diabetes. *Neurobiology of Disease*.

[89] Schmeichel AM, Schmelzer JD, Low PA (2003). Oxidative injury and apoptosis of dorsal root ganglion neurons in chronic experimental diabetic neuropathy. *Diabetes*.

[90] Kamiya H, Zhang W, Sima AAF (2005). Apoptotic stress is counter balanced by survival elements preventing programmed
cell death of dorsal root Ganglions in subacute type 1 diabetic BB/Wor-rats. *Diabetes*.

[91] Cheng C, Zochodne DW (2003). Sensory neurons with activated caspase-3 survive long-term experimental diabetes. *Diabetes*.

[92] Kamiya H, Zhang W, Sima AAF (2006). Degeneration of the Golgi and neuronal loss in dorsal root ganglia in diabetic
BB/Wor-rats. *Diabetologia*.

[93] Thomas PK, Beamish NG, Small JR (1996). Paranodal structure in diabetic sensory polyneuropathy. *Acta Neuropathologica*.

[94] Dyck PJ, Giannini C (1996). Pathologic alterations in the diabetic neuropathies of humans: a review. *Journal of Neuropathology & Experimental Neurology*.

[95] Sima AAF (1997). Diabetic polyneuropathy (Letter to the Editor). *Journal of Neuropathology & Experimental Neurology*.

[96] Brismar T, Sima AAF, Greene DA (1987). Reversible and irreversible nodal dysfunction in diabetic neuropathy. *Annals of Neurology*.

[97] Cherian PV, Kamijo M, Angelides KJ, Sima AAF (1996). Nodal Na^+^ -channel displacement is associated with nerve-conduction slowing in the
chronically diabetic BB/W-rat: prevention by aldose reductase inhibition. *Journal of Diabetes and Its Complications*.

[98] Sima AAF, Cherian PV (1997). Neuropathology of diabetic neuropathy and its correlations with neurophysiology. *Clinical Neuroscience*.

[99] Sima AAF, Zhang W, Li Z-G, Murakawa Y, Pierson CR (2004). Molecular alterations underlie nodal and paranodal degeneration in type 1 diabetic neuropathy and are prevented by C-peptide. *Diabetes*.

[100] Sugimoto K, Murakawa Y, Zhang W, Xu G, Sima AAF (2000). Insulin receptor in rat peripheral nerve: its localization and alternatively spliced isoforms. *Diabetes/Metabolism Research and Reviews*.

[101] Li Z-G, Zhang W, Sima AAF (2003). C-peptide enhances insulin-mediated cell growth and protects against high glucose induced apoptosis. *Brain Path*.

[102] Forst T, Dufayet de la Tour D, Kunt T (2000). Effects of proinsulin C-peptide on nitric oxide, microvascular blood flow and erythrocyte Na^+^, K^+^ -ATPase activity in diabetes mellitus type I. *Clinical Science*.

[103] Kunt T, Schneider S, Pfützner A (1999). The effect of human proinsulin C-peptide on erythrocyte deformability in patients
with type I diabetes mellitus. *Diabetologia*.

[104] Ohtomo Y, Aperia A, Sahlgren B, Johansson B-L, Wahren J (1996). C-peptide stimulates rat renal tubular Na^+^, K^+^-ATPase activity in synergism with neuropeptide Y. *Diabetologia*.

[105] Zhong Z, Davidescu A, Ehrén I (2005). C-peptide stimulates ERK 1/2 and JNK MAP kinases via activation of protein kinase C in human renal tubular cells. *Diabetologia*.

[106] Pop-Busi R, Sima AAF, Stevens M (2006). Diabetic neuropathy and oxidative stress. *Diabetes/Metabolism Research and Reviews*.

[107] Cameron NE, Cotter MA, Archibald V, Dines KC, Maxfield EK (1994). Anti-oxidant and pro-oxidant effects on nerve conduction velocity, endoneurial blood flow and oxygen tension in non-diabetic and streptozotocin-diabetic rats. *Diabetologia*.

[108] Cameron NE, Cotter MA, Sima AAF (1997). Oxidative stress and abnormal lipid metabolism in diabetic complication. *Chronic Complications in Diabetes: Animal Models and Chronic Complications*.

[109] Requena JR, Baynes JW, Sima AAF (1999). Studies in animal models on the role of glycation and advanced glycation end products (AGE's) in the pathogenes of diabetic complications: pitfalls and limitations. *Chronic Complications in Diabetes: Animal Models and Chronic Complications*.

[110] Wallerath T, Kunt T, Forst T (2003). Stimulation of endothelial nitric oxide synthase by proinsulin C-peptide. *Nitric Oxide*.

[111] Kitamura T, Kimura K, Makondo K (2003). Proinsulin C-peptide increases nitric oxide production by enhancing mitogen-activated protein kinase-dependent transcription of endothelial nitric oxide synthase in aortic endothelial cells of Wistar rats. *Diabetologia*.

[112] Scalia R, Coyle K, Levine B, Booth G, Lefer A (2000). C-peptide inhibits leucocyte-endothelium interaction in the microcirculation during endothelial dysfunction. *The FASEB Journal*.

[113] Li H, Xu L, Dunbar JC, Dhabuwala CB, Sima AAF (2004). Effects of C-peptide on expression of eNOS and iNOS in human cavernosal smooth muscle cells^⋆^1. *Urology*.

[114] Cotter MA, Ekberg K, Wahren J, Cameron NE (2003). Effects of proinsulin C-peptide in experimental diabetic neuropathy:
vascular actions and modulation by nitric oxide synthase inhibition. *Diabetes*.

[115] Li Z-G, Zhang W, Sima AAF (2003). C-peptide enhances insulin-mediated cell growth and protection against high
glucose-induced apoptosis in SH-SY5Y cells. *Diabetes/Metabolism Research and Reviews*.

[116] Li Z-G, Zhang W, Sima AAF (2005). The role of impaired insulin/IGF action in primary diabetic encephalopathy. *Brain Research*.

[117] Li Z-G, Zhang W, Sima AAF (2002). C-peptide prevents hippocampal apoptosis in type 1 diabetes. *International Journal of Experimental Diabetes Research*.

[118] Pierson CR, Zhang W, Sima AAF (2003). Proinsulin C-peptide replacement in type 1 diabetic BB/Wor-rats prevents deficits in nerve fiber regeneration. *Journal of Neuropathology & Experimental Neurology*.

[119] Kamiya H, Zhang W, Sima AAF (2005). Apoptotic stress is counterbalanced by survival elements preventing programmed cell death of dorsal root ganglions in subacute type 1 diabetic BB/Wor-rats. *Diabetes*.

[120] Yamamoto K, Merry AC, Sima AAF (1996). An orderly development of paranodal axoglial junctions and bracelets of Nageotte in the rat sural nerve. *Developmental Brain Research*.

[121] Kramer L, Fasching P, Madl C (1998). Previous episodes of hypoglycemic coma are not associated with permanent cognitive brain dysfunction in IDDM patients on intensive insulin treatment. *Diabetes*.

[122] Ott A, Stolk RP, van Harskamp F, Pols HAP, Hofman A, Breteler MMB (1999). Diabetes mellitus and the risk of dementia: the Rotterdam study. *Neurology*.

[123] Arvanitakis Z, Wilson RS, Bievas JL, Evans DA, Bennett DA (2004). Diabetes mellitus and risk of Alzheimer's disease and decline in cognitive function. *Archives of Neurology*.

[124] Schoenle EJ, Schoenle D, Mokiosari L, Largo RH (2002). Impaired intellectual development in children with type I diabetes: association
with HbA1c, age at diagnosis and sex. *Diabetologia*.

[125] Ryan CM (1988). Neurobehavioral complications of type 1 diabetes. Examination of possible risk factors. *Diabetes Care*.

[126] Sima AAF, Kamiya H, Li Z-G (2004). Insulin, C-peptide, hyperglycemia, and central nervous system complications in diabetes. *European Journal of Pharmacology*.

[127] Kurita A, Mochio S, Isogai Y (1995). Changes in auditory P300 event-related potentials and brainstem evoked potentials in diabetes mellitus. *Acta Neurologica Scandinavica*.

[128] Holmes CS, Richman LC (1985). Cognitive profiles of children with insulin-dependent diabetes. *Journal of Developmental and Behavioral Pediatrics*.

[129] Lunetta M, Damanti AR, Fabbri G, Lombardo M, Di Mauro M, Mughini L (1994). Evidence by magnetic resonance imaging of cerebral alterations of atrophy type in young insulin-dependent diabetic patients. *Journal of Endocrinological Investigation*.

[130] Perros P, Deary IJ, Sellar RJ, Best JJK, Frier BM (1997). Brain abnormalities demonstrated by magnetic resonance imaging in adult IDDM patients with and without a history of recurrent severe hypoglycemia. *Diabetes Care*.

[131] Dey J, Misra A, Desai NG, Mahapatra AK, Padma MV (1997). Cognitive function in younger type II diabetes. *Diabetes Care*.

[132] Ryan CM (2006). Why is cognitive dysfunction associated with the development of diabetes early in life? The diathesis hypothesis. *Pediatric Diabetes*.

[133] Kamal A, Biessels G-J, Urban IJA, Gispen WH (1999). Hippocampal synaptic plasticity in streptozotocin-diabetic rats: impairment of long-term potentiation and facilitation of long-term depression. *Neuroscience*.

[134] Biessels G-J, Kamal A, Urban IJA, Spruijt BM, Erkelens DW, Gispen WH (1998). Water maze learning and hippocampal synaptic plasticity in streptozotocin-diabetic rats: effects of insulin treatment. *Brain Research*.

[135] Li Z-G, Zhang W, Grunberger G, Sima AAF (2002). Hippocampal neuronal apoptosis in type 1 diabetes. *Brain Research*.

[136] Sima AAF, Li Z-G (2005). The effect of C-peptide on cognitive dysfunction and hippocampal apoptosis in type 1 diabetic rats. *Diabetes*.

